# Machine Learning-Based Prediction of Institutional Delivery Dropout (IDD) Among Nigerian Women: An Exploratory Study Using SHAP Interpretability

**DOI:** 10.1007/s44197-026-00525-y

**Published:** 2026-02-23

**Authors:** Jamilu Sani, Anas Ali Alhur, Mohamed Mustaf Ahmed

**Affiliations:** 1 Department of Demography and Social Statistics, Federal University Birnin Kebbi, Birnin Kebbi, Kebbi State Nigeria; 2 Department of Health Informatics, College of Public Health and Health Informatics, University of Hail, Hail, Saudi Arabia; 3 Faculty of Medicine and Health Sciences, SIMAD University, Mogadishu, Somalia

**Keywords:** Machine learning, Institutional delivery dropout, Maternal health, Antenatal care, SHAP explanations, Support vector machine, Nigeria, Sociodemographic determinants

## Abstract

**Introduction:**

Institutional delivery dropout (IDD), defined as delivery outside a health facility despite attending antenatal care (ANC), remains a significant barrier to reducing maternal mortality in Nigeria. Traditional statistical models often fall short of capturing the complex, non-linear interactions among the socio-demographic factors that drive this critical health behavior.

**Methods:**

Using a comprehensive dataset of 16,100 women from the 2018 Nigeria Demographic and Health Survey (NDHS), we applied and compared seven diverse machine learning (ML) algorithms, including models such as Support Vector Machine (SVM), Gradient Boosting (GB), and Extreme Gradient Boosting (XGBoost). The model performance was systematically evaluated using metrics such as accuracy, Area Under the Receiver Operating Characteristic curve (AUROC), F1-score, and detailed confusion matrices. Furthermore, SHapley Additive explanations (SHAP) were used to provide transparent interpretations of feature importance and predictive contributions.

**Results:**

Gradient Boosting was the best-performing model, achieving the highest F1-score (0.755) and AUROC (0.82). SVM achieved the highest accuracy (0.740) and recall (0.780). SHAP identified education level, household wealth, and religion as strong predictors of IDD. The performance metrics reported with confidence intervals showed modest variability across the models.

**Conclusion:**

Machine learning approaches were effective in identifying women at an increased risk of institutional delivery dropout. SHAP analysis provides insights into the key sociodemographic predictors of IDD, highlighting the value of interpretable ML methods in maternal health research.

## Introduction

Ensuring skilled attendance at birth is a critical strategy for reducing both maternal and neonatal mortalities, particularly in low- and middle-income countries (LMICs) such as Nigeria (1, 2). Despite remarkable gains in antenatal care (ANC) coverage across sub-Saharan Africa, many women who attend ANC still deliver outside health facilities, a phenomenon referred to as institutional delivery dropout (IDD) (3, 4). This discontinuity in the maternal healthcare continuum undermines the potential benefits of ANC and remains a persistent challenge in achieving Sustainable Development Goal 3.1, which targets a substantial reduction in maternal mortality (5). Studies have examined the determinants of institutional delivery using conventional statistical approaches, primarily logistic regression and multivariable analysis (6, 7, 8, 9, 10). While these methods are valuable for identifying average effects and testing associations by often relying on linear assumptions, they may struggle to capture complex non-linear interactions between predictors, thereby providing limited utility for individual-level risk prediction (11).

As the global health landscape increasingly embraces data-driven approaches, there is growing interest in leveraging machine learning (ML) techniques to augment traditional analytics in maternal health research (12, 13, 14, 15). Machine learning offers a flexible, data-driven alternative that prioritizes robust predictions over strict causal inference. Rather than solely testing pre-defined hypotheses about associations, ML models aim to optimize predictive performance on unseen data, making them particularly suited for identifying women at high risk of IDD based on intricate patterns in sociodemographic data (16, 17). Moreover, recent advances in explainable AI (XAI), such as SHapley Additive exPlanations (SHAP), have addressed the traditional “black-box” interpretability gap in many ML models, enabling stakeholders to understand the contribution of individual features to specific model predictions (18, 19, 20). While existing studies have applied ensemble machine learning with SHAP interpretation to maternal health outcomes, particularly in modeling early ANC initiation in Nigeria and sub-Saharan Africa (21, 22, 23), few have examined the specific discontinuity between ANC attendance and institutional deliveries. Limited research has focused specifically on IDD, which represents a distinct behavioral discontinuity following ANC attendance and reflects the barriers encountered after initial engagement with the health system. This gap is underexplored in maternal health analytics and requires targeted investigations.

Moreover, few studies have systematically compared multiple ML algorithms to predict IDD or integrate SHAP to interpret the influence of individual predictors in the Nigerian context. Our study addresses this gap by examining dropout behavior after ANC uptake and applying multiple ML classifiers alongside SHAP explanations to uncover the key sociodemographic drivers of IDD.

Despite the growing body of literature applying ML in health research, few studies have specifically explored its application in predicting institutional delivery dropouts, particularly in the Nigerian context. Given Nigeria’s substantial maternal mortality burden and persistent regional disparities in healthcare access, predictive modeling offers an opportunity to improve identification of women at elevated risk of IDD (21, 22, 24, 25). More recent work has introduced ensemble machine learning to maternal health, for example, predicting early antenatal care initiation (21, 22, 23); however, these efforts were generally limited by smaller datasets or a narrow methodological scope. Recent ML-based studies have further extended these approaches to a variety of maternal and reproductive health outcomes, such as mode of delivery prediction (26, 27), fertility preferences (22, 28), and maternal risk stratification using sparse clinical and demographic data (29, 30, 31).

These advancements highlight the growing potential of ML to complement conventional epidemiologic analysis; however, few have focused explicitly on IDD, which represents a distinct discontinuity after ANC attendance. The drawbacks of existing studies include (i) reliance on a single algorithm without systematic benchmarking, (ii) limited interpretability of predictions, and (iii) insufficient analysis of the ANC-to-delivery discontinuity. In addition, earlier research seldom incorporated SHAP or related XAI techniques to enhance interpretability and policy translations. Our study addresses these gaps by comparing seven diverse classifiers on a large national dataset and employing SHAP to make predictions interpretable and actionable. Therefore, this study seeks to fill these gaps by systematically comparing seven supervised machine learning algorithms, including ensemble models such as Gradient Boosting and XGBoost, using large, nationally representative datasets. It also employs SHAP explanations to enhance interpretability and generate transparent, feature-level insights into the sociodemographic determinants of institutional delivery dropouts. The major contributions of this study are threefold: (i) it introduces a multi-model ML framework for predicting institutional delivery dropout among Nigerian women who attended ANC; (ii) it benchmarks model performance across diverse classifiers to identify the most effective predictive model; and (iii) it integrates SHAP explanations to interpret the relative importance of predictors, thereby enhancing policy relevance and model transparency.

## Methods

### Study Design and Data Source

This analysis was based on a cross-sectional study design utilizing data from the 2018 Nigeria Demographic and Health Survey (NDHS). The NDHS is a nationally representative survey coordinated by the National Population Commission in partnership with the Inner City Fund (ICF) and covers all six geopolitical zones of Nigeria. A multistage stratified cluster sampling approach ensured comprehensive representation of rural and urban populations and various socioeconomic strata. For this study, we extracted data from the Individual Recode (IR) file, which contains detailed information on women’s reproductive health and health care utilization. The IR file was selected because it provides individual-level data on women who had a live birth within five years preceding the survey, allowing for an objective linkage between ANC attendance and place of delivery. This ensured the completeness and consistency of the variables used for the institutional delivery dropout analysis. In this study, institutional delivery dropout is conceptualized as a maternal-level behavioral outcome reflecting a woman’s delivery decision following engagement with antenatal care services. Accordingly, women were treated as the unit of analysis. While the Birth Recode (BR) file is commonly used for child-level outcomes in DHS analyses, the IR file is appropriate for research questions focused on women’s healthcare utilization and decision-making, particularly when the outcome reflects post-ANC delivery behavior rather than child-specific events.

### Study Population

**Fig. 1:**
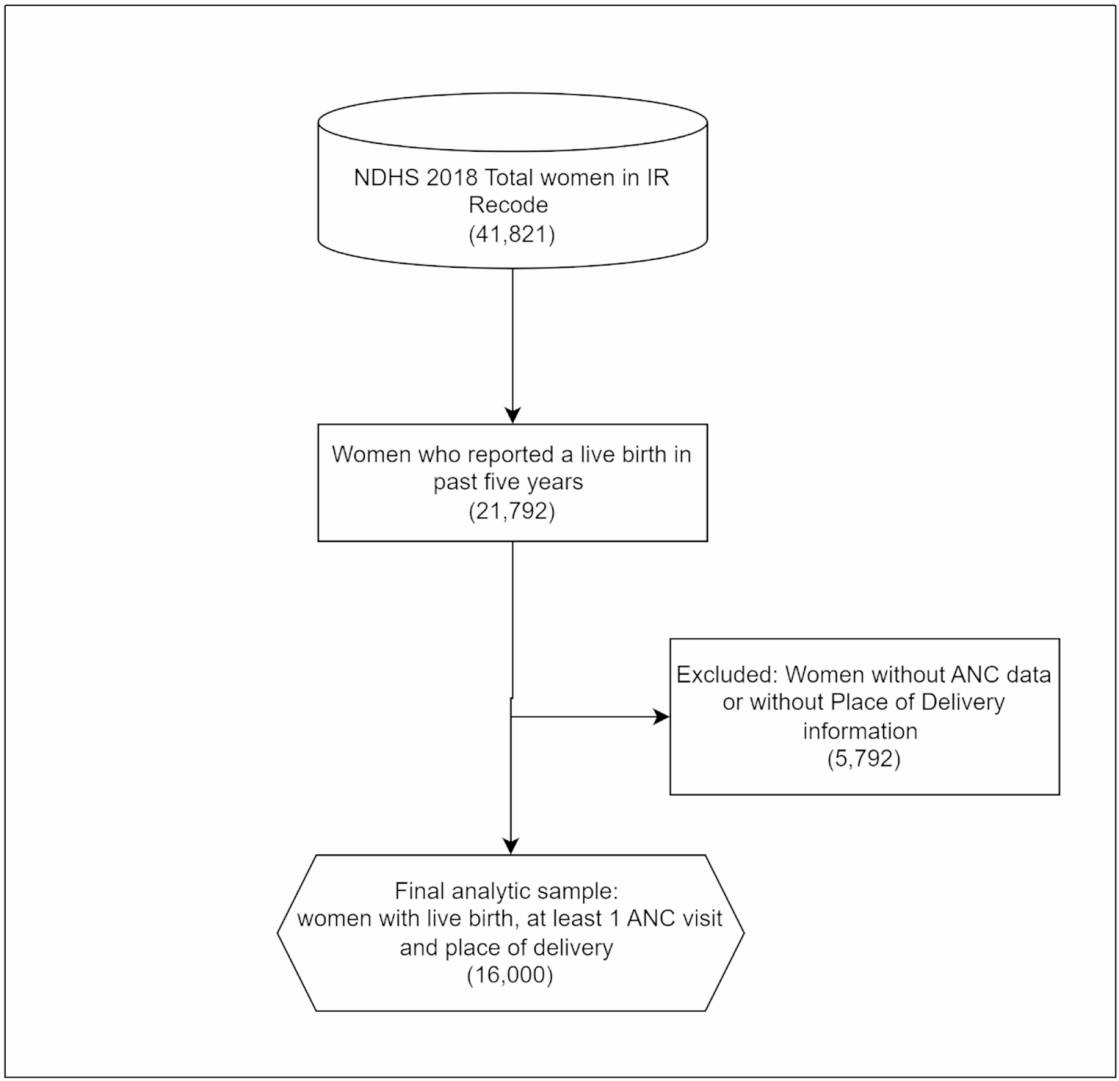
Flow diagram showing the selection of the final analytic sample from the 2018 Nigeria Demographic and Health Survey (NDHS). The final sample included women who had a live birth within five years, attended at least one antenatal care visit, and reported their delivery location

The study population comprised women aged 15–49 years who had experienced a live birth within five years preceding the survey and had attended at least one ANC visit during that pregnancy. These women were also required to report their place of delivery. From the 41,821 women interviewed in the NDHS Individual Recode file, 21,792 reported having a live birth in the five years before the survey. Women with missing information on ANC attendance or place of delivery were excluded, resulting in a final unweighted analytic sample of 16,000 women. This subpopulation appropriately represents women in contact with the health system through ANC and thus aligns with the study objective of predicting institutional delivery dropout. A schematic flow diagram summarizing the sample-selection process is presented in Fig. 1. The analysis was restricted to one observation per woman, corresponding to the most recent live birth within the five years preceding the survey, to ensure independence of observations and a clear temporal linkage between antenatal care attendance and place of delivery. While DHS birth history analyses commonly adopt a child-based unit of analysis for outcomes such as fertility and birth intervals, institutional delivery dropout reflects a delivery decision made by the woman following ANC attendance. Therefore, a woman-level analytical approach was appropriate for the predictive objectives of this study.

### Outcome Variable

The outcome of interest was IDD, defined as non-utilization of a health facility for delivery despite attending one or more ANC visits. This was operationalized as a binary variable: “1” indicating institutional delivery dropout (delivery outside a recognized health facility despite ANC attendance) and “0” indicating institutional delivery (delivery in a recognized public or private health facility).

### Predictor Variables

The analysis included a broad range of sociodemographic and contextual variables, informed by prior research and data availability. These included maternal age, educational attainment, wealth index, employment status, marital status, religious affiliation, perceived difficulty in accessing health facilities, place of residence (urban/rural), and geopolitical region. These factors were selected because of their known associations with maternal health behaviors and service utilization patterns (3, 6, 7, 9, 10, 21, 22). Self-reported obstetric experience variables, such as delivery complications, were excluded because they were inconsistently reported and exhibited substantial missingness. Facility-level characteristics, including staffing levels, service readiness, and objective quality-of-care indicators, were not collected in the DHS household survey and were therefore unavailable for inclusion. This analytic focus on population-level sociodemographic predictors improves generalizability while avoiding bias arising from incomplete or unavailable variables. Following data screening and feature selection, ten (10) sociodemographic predictors were retained for the final machine learning models.

### Variable Coding and Recategorization

All variables were coded following the standard Demographic and Health Survey (DHS) recode definitions and prior maternal health literature. The outcome variable, institutional delivery dropout [IDD], was constructed by combining information on antenatal care attendance and place of delivery and was coded as a binary indicator, where 1 = institutional delivery dropout (delivery outside a recognized health facility despite attending at least one ANC visit) and 0 = institutional delivery (delivery in a recognized public or private health facility). Maternal education was categorized into four levels (none, primary, secondary, and higher). Household wealth was classified using the DHS wealth index quintiles. Maternal age was grouped into five-year categories to capture potential non-linear effects. Other categorical variables, including religion, marital status, place of residence, employment status, perceived difficulty in accessing health facilities, and geopolitical regions, were coded using standard DHS classifications. These coding procedures were consistently applied across all models to ensure comparability and reproducibility.

### Data Processing and Analytical Approach

Initial data handling and exploratory analyses were conducted using STATA 17 to generate descriptive statistics, bivariate associations, and crude odds ratios (CORs) with 95% confidence intervals (CIs). Descriptive analyses applied DHS weights, and bivariate associations were tested using chi-square tests, with statistical significance set at *p* < 0.05. Although the ML models were not survey-weighted, their outputs were benchmarked against epidemiological measures to ensure consistency. Missing data were examined for all candidate variables. Variables with substantial missingness (e.g., facility-level indicators) were excluded to avoid bias, while sociodemographic predictors with negligible item non-response (< 2%) were retained for the complete case analysis. This approach-maintained data integrity while avoiding potential distortions from imputing categorical sociodemographic variables. All predictors were categorical and had complete responses, requiring only standard label encoding for ML implementation. The descriptive and bivariate analyses conducted in STATA 17 served an inferential purpose, whereas the machine learning analyses were explicitly predictive.

Subsequent analyses, including model development and evaluation, were performed using Python (v3.10) in a Jupyter Notebook environment. Although descriptive and bivariate statistics used DHS sampling weights to maintain epidemiological validity, the ML models did not incorporate these weights. This decision was made because of the inherent methodological limitations of directly applying complex survey designs (e.g., stratification, clustering, and weighting) within standard ML pipelines. The primary objective was to maximize predictive performance and identify underlying patterns in IDD behavior, rather than generating nationally representative point estimates that require survey-weighted analyses. This approach is consistent with prior ML studies using DHS data, which benchmark predictive accuracy rather than population inferences. Accordingly, formal statistical significance testing of the model performance differences (e.g., DeLong or McNemar tests) was not conducted, as the analysis was not intended to support inferential claims about model superiority. To quantify the uncertainty in model performance, stratified bootstrapping with 1,000 resamples was used to compute 95% confidence intervals for test set accuracy, F1-score, and AUROC. In addition, the 5-fold cross-validation performance was summarized using the mean and standard deviation across the folds.

### Platform and Implementation

All models were implemented using Python 3.10 with Scikit-learn v1.2.2, SHAP v0.41.0, and Seaborn v0.12.2. A random seed of 42 was used to ensure reproducibility. Although descriptive and bivariate analyses used DHS sampling weights to maintain epidemiologic validity, the machine learning models did not incorporate these weights. This decision reflects the methodological limitations of applying complex survey features (such as stratification, clustering, and weighting) within the standard machine learning pipelines. The primary objective of the ML analysis was to maximize predictive performance and identify underlying patterns in IDD behavior, rather than producing nationally representative estimates requiring survey-weighted procedures. This non-weighted approach is consistent with prior ML applications using DHS data, which focus on model behavior and interpretability rather than population-level inference. Model calibration was assessed using calibration curves and Brier scores to evaluate how well the predicted probabilities aligned with the observed outcomes. The procedures are described here, and the calibration results are presented in the Results section.

### Research Questions

This study was guided by two primary research questions: (i) Which machine learning model best predicts institutional delivery dropout among Nigerian women based on the 2018 NDHS dataset? (ii) Which sociodemographic factors and predictors most strongly influence institutional delivery dropout, and how do they contribute to the model’s predictions?

### Feature Selection Strategy

**Fig. 2:**
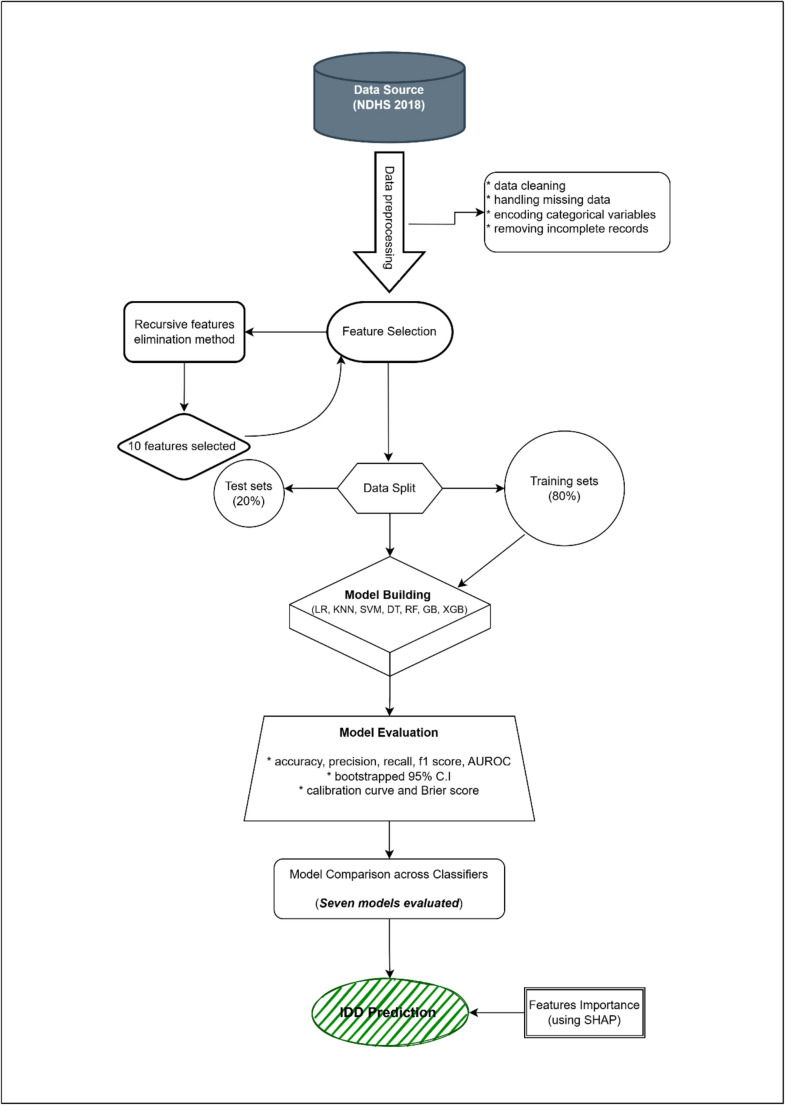
Flowchart illustrating the key stages of the machine learning pipeline for predicting IDD

Exploratory data analysis (EDA) and statistical screening guided the selection of the initial variables (Fig. 2). To assess multicollinearity and ensure a stable feature set for model training, Cramér’s V statistic was used for the categorical predictors. Subsequently, Recursive Feature Elimination (RFE) with a Random Forest estimator was employed to iteratively identify the most predictive variables, aiming to optimize the model performance and simplify the interpretability. This method was selected for its ability to effectively handle categorical predictors and prevent overfitting while maintaining interpretability. Ten predictors were ultimately retained based on the RFE rankings and consistency with the epidemiological evidence.

### Model Development and Evaluation

Our machine learning approach for predicting IDD involved a systematic pipeline, as shown in Fig. 1. Seven machine learning classifiers were trained to predict IDD: Logistic Regression (LR), Support Vector Machine (SVM), K-Nearest Neighbors (KNN), Decision Tree (DT), Random Forest (RF), Gradient Boosting (GB), and XGBoost (XGB). The dataset was split into training (80%) and test (20%) subsets, which were stratified by the outcome variable IDD to ensure representative proportions in both the sets. Stratified 5-fold cross-validation was used during model training to enhance generalizability and reduce overfitting. The default hyperparameters were applied uniformly across the models to ensure a fair and consistent benchmarking framework. This choice reflects a deliberate focus on baseline model comparison rather than hyperparameter optimization for the deployment. This was appropriate for an exploratory analysis focused on comparing baseline algorithmic behavior rather than producing optimized models for deployment. The model performance was evaluated using accuracy, precision, recall, F1-score, AUROC, confusion matrices, and calibration metrics. Best-practice evaluation relied on probability-based AUROC values and uncertainty estimates derived from bootstrapped confidence intervals and cross-validation variabilities.

### Model Interpretation and Feature Importance

To gain insight into the drivers of model predictions, we computed SHAP (SHapley Additive exPlanations) values. Although Gradient Boosting achieved the highest overall predictive performance, SHAP values were computed using the Random Forest model because of its robust performance and enhanced interpretability, particularly for feature-level explanations. The feature importance patterns were consistent across the models. SHAP decomposes model outputs and quantifies the contribution of each feature to predicting institutional delivery dropouts. We used both global summaries (mean SHAP values) and individual feature impact plots to interpret the model behavior and rank the predictors by their influence.

Partial dependence and ICE plots were considered but not implemented because SHAP adequately captured the feature effects within the study’s exploratory scope. SHAP was deemed sufficient for interpretability in this context, as it provides consistent, model-agnostic explanations suitable for both linear and ensemble models, aligning with the study’s focus on transparency and policy-relevant interpretation rather than exhaustive model explainability.

## Results

### Sociodemographic Characteristics and Predictors of IDD

Table 1 presents the sociodemographic characteristics of the 16,100 women analyzed, along with their associations with IDD. A substantial dropout rate was observed, with nearly half of the women (48%) delivering outside health facilities despite having attended at least one ANC visit. Regarding age distribution, most respondents were between 25 and 29 years (26.38%), followed by 30–34 years (22.88%) and 20–24 years (18.76%). The youngest group (15–19 years) comprised the smallest proportion (4.98%). IDD was lowest among the youngest age group (38.4%) and progressively increased with age, reaching a peak of 58.0% among women aged 35–39 years old. Crude odds ratios (CORs) confirmed that older age groups had significantly higher odds of IDD than those aged 15–19 years, with women aged 35–39 years showing more than twice the odds (COR: 2.22; 95% CI: 1.83–2.68).

Educational attainment was strongly associated with the utilization of institutional delivery. While one-third of the women had no formal education (33.92%), only 11.29% had higher education. Dropout rates were highest among women with no education (76.1%) and declined sharply with increasing educational levels, dropping to 11.9% among those with higher education. The likelihood of IDD was significantly lower among educated women; those with higher education had 96% lower odds of dropout compared with women with no education (COR: 0.04; 95% CI: 0.035–0.051). Marital status also influences delivery choices. Most women were married (90.78%); however, women who were never in union or cohabiting showed higher institutional delivery uptake than married women. Compared with women who were never in union, married women were significantly less likely to use institutional deliveries (COR: 0.59; 95% CI: 0.47–0.73). Religion was another significant factor: 72.2% of Christian women used institutional delivery compared with only 36.2% of Muslim women. Consequently, Muslim women had substantially lower odds of institutional delivery (COR: 0.22; 95% CI: 0.20–0.24) than their Hindu counterparts.

Wealth status revealed a pronounced socioeconomic gradient. Women in the richest quintile had the lowest dropout rate (18.8%) and were 18 times more likely to use institutional delivery than those in the poorest quintile (COR: 17.85; 95% CI: 15.24–20.91). Similarly, women who reported that distance to health facilities was not a major problem were significantly more likely to complete an institutional delivery (COR: 1.33; 95% CI: 1.22–1.45). Employment status, place of residence, and geopolitical regions also showed notable associations. Employed women were more likely to deliver at health facilities (COR: 1.74; 95% CI: 1.60–1.89). Urban residence strongly favored institutional delivery; women in rural areas had significantly lower odds of institutional delivery than those in urban areas (COR: 0.30; 95% CI: 0.28–0.33). Regionally, the Southeast (82.9%) and Southwest (79.3%) had the highest institutional delivery rates, while the Northwest recorded the lowest (24.1%). Women in the South East had over 15 times higher odds of institutional delivery than those in the North West (COR: 15.33; 95% CI: 13.28–17.69).

**Table 1:** Sociodemographic characteristics and association of predictors with outcome (*N* = 16,100)

Variable	Overall *n* (%)	Institutional Delivery		COR (95% CI)
		NO - IDD (%)	YES (%)	
Age Group				
15–19 (ref.)	802 (4.98)	494 (61.60)	308 (38.40)	1
20–24	3,019 (18.76)	1,649 (54.61)	1,371 (45.39)	1.33 (1.11, 1.61) **
25–29	4,247 (26.38)	2,007 (47.26)	2,240 (52.74)	1.79 (1.49, 2.15) ***
30–34	3,684 (22.88)	1,626 (44.15)	2,058 (55.85)	2.03 (1.69, 2.44) ***
35–39	2,683 (16.67)	1,127 (41.99)	1,557 (58.01)	2.22 (1.83, 2.68) ***
40–44	1,193 (7.41)	572 (47.94)	621 (52.06)	1.74 (1.41, 2.16) ***
45–49	471 (2.92)	239 (50.82)	232 (49.18)	1.55 (1.19, 2.02) ***
**Educational Level**				
No Education (ref.)	5,461 (33.92)	4,156 (76.10)	1,305 (23.90)	1
Primary	2,674 (16.61)	1,397 (52.27)	1,277 (47.73)	2.91 (2.58, 3.27) ***
Secondary	6,147 (38.18)	1,944 (31.63)	4,203 (68.37)	6.88 (6.25, 7.58) ***
Higher	1,818 (11.29)	217 (11.93)	1,601 (88.07)	23.50 (19.52, 28.28) ***
**Marital Status**				
Never in Union (ref.)	380 (2.36)	137 (35.99)	243 (64.01)	1
Married	14,615 (90.78)	7,159 (48.98)	7,457 (51.02)	0.59 (0.47, 0.73) ***
Living with Partner	512 (3.18)	183 (35.76)	329 (64.24)	1.01 (0.75, 1.35)
Widowed	209 (1.3)	74 (35.69)	134 (64.31)	1.01 (0.69, 1.49)
Divorced/Separated	384 (2.39)	162 (42.03)	223 (57.97)	0.78 (0.56, 1.07)
**Religion**				
Christianity (ref.)	7,115 (44.19)	1,982 (27.85)	5,133 (72.15)	1
Islam	8,910 (55.34)	5,688 (63.83)	3,222 (36.17)	0.22 (0.20, 0.24) ***
Others	75 (0.47)	45 (59.93)	30 (40.07)	0.26 (0.16, 0.41) ***
**Wealth Index**				
Poorest (ref.)	2,434 (15.12)	1,961 (80.56)	473 (19.44)	1
Poorer	3,102 (19.27)	2,088 (67.31)	1,014 (32.69)	2.01 (1.76, 2.30) ***
Middle	3,516 (21.84)	1,777 (50.55)	1,738 (49.45)	4.05 (3.56, 4.62) ***
Richer	3,563 (22.13)	1,231 (34.56)	2,332 (65.44)	7.85 (6.84, 9.02) ***
Richest	3,485 (21.65)	657 (18.84)	2,828 (81.16)	17.85 (15.24, 20.91) ***
**Distance to Health Facility**				
Big problem (Ref)	1,975 (12.27)	1,075 (54.43)	900 (45.57)	1
Not a big problem	14,125 (87.73)	6,640 (47.01)	7,485 (52.99)	1.33 (1.22–1.45) ***
**Working Status**				
No (ref.)	4,442 (27.59)	2,569 (57.83)	1,873 (42.17)	1
Yes	11,658 (72.41)	5,146 (44.14)	6,513 (55.86)	1.74 (1.60, 1.89) ***
**Residence**				
Urban (ref.)	7,507 (46.63)	2,433 (32.40)	5,075 (67.60)	1
Rural	8,593 (53.37)	5,282 (61.47)	3,311 (38.53)	0.30 (0.28, 0.33) ***
**Region**				
North West (ref.)	4,824 (29.96)	3,663 (75.93)	1,161 (24.07)	1
North Central	2,156 (13.39)	756 (35.07)	1,400 (64.93)	5.84 (5.18, 6.59) ***
North East	2,738 (17)	1,747 (63.81)	991 (36.19)	1.79 (1.59, 2.02) ***
South East	2,009 (12.48)	343 (17.07)	1,666 (82.93)	15.33 (13.28, 17.69) ***
South South	1,557 (9.67)	622 (39.98)	934 (60.02)	4.74 (4.11, 5.45) ***
South West	2,816 (17.49)	583 (20.69)	2,233 (79.31)	12.09 (10.28, 14.23) ***

### Machine Learning Model Performance Evaluation

**Fig. 3:**
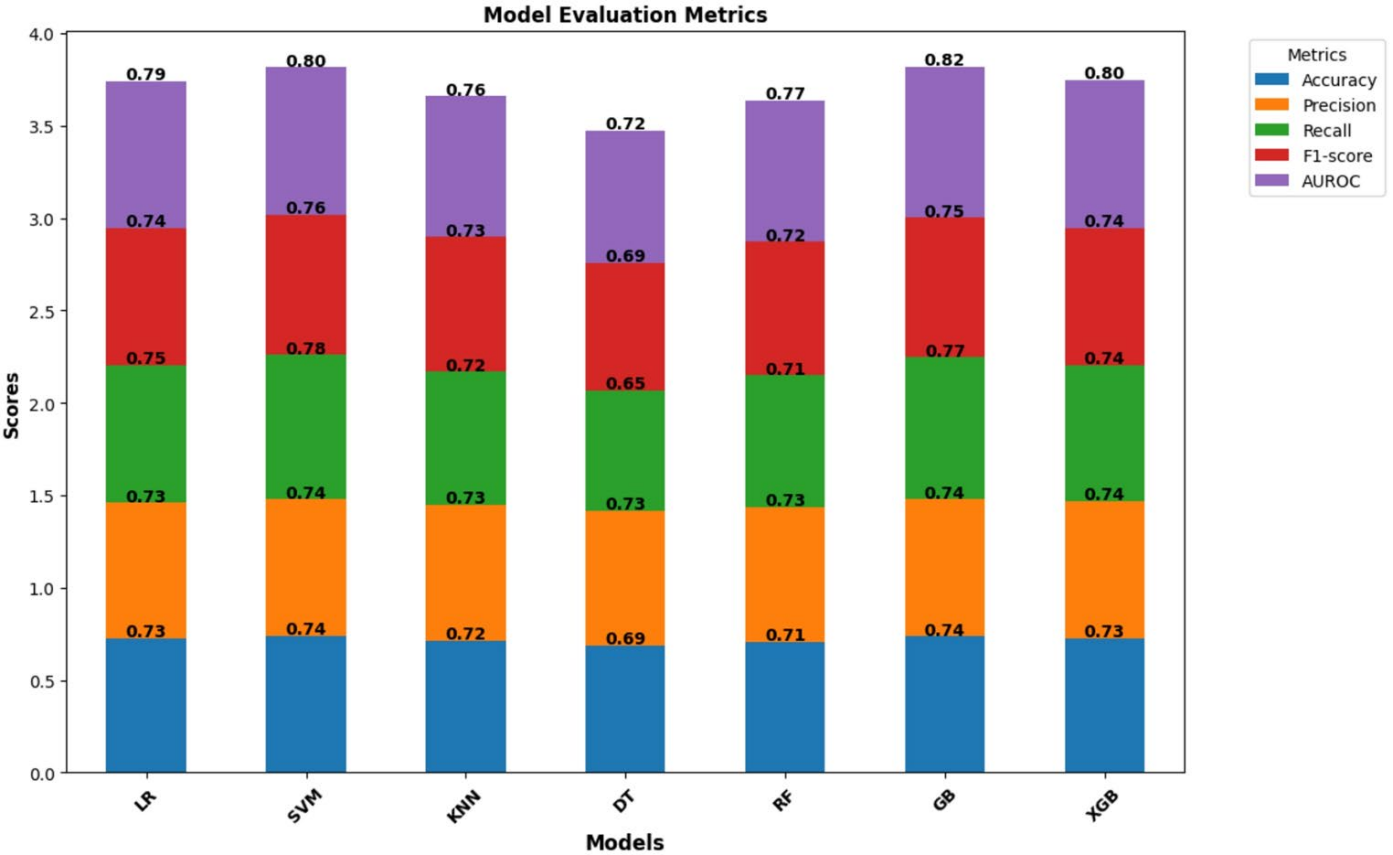
Comparison of Model Performance Metrics

Table 2; Fig. 3 summarize the performances of the seven machine learning algorithms. Given the 48% prevalence of institutional delivery dropout, accuracy as a performance metric may be misleading because it does not account for imbalanced class distributions. Therefore, additional metrics such as precision, recall, F1-score, and AUROC were reported to provide a more nuanced assessment of the model performance. Among all the models, SVM achieved the highest recall (0.780), confirming its strength in minimizing false negatives and identifying women at risk of dropout. Gradient Boosting achieved the highest AUROC (0.82; 95% C.I: 0.805–0.831), indicating the strongest overall discrimination between dropout and non-dropout. Ensemble models, such as Gradient Boosting and XGBoost, outperformed simpler classifiers, demonstrating a better capacity to capture nonlinear relationships in the predictors.

SVM achieved a competitive accuracy of 0.740 and an F1-score of 0.759 (95% C.I: 0.742–0.775), ranking closely behind Gradient Boosting. Gradient Boosting achieved the highest F1-score (0.755; 95% CI: 0.738–0.769) and strong precision (0.740), reflecting balanced and stable predictive performance across metrics. GB also showed strong performance in terms of accuracy (0.737) and recall (0.740). XGBoost also achieved high precision (0.741); however, its overall accuracy (0.727) and F1-score (0.739) were slightly lower than those of SVM and GB. Decision Tree and KNN models demonstrated comparatively lower performance across metrics, consistent with their known sensitivity to noise and limited ability to model complex interactions. Similarly, the LR model also showed moderate performance (accuracy of 0.725 and AUROC of 0.794).

Cross-validation performance further supported these patterns, with Gradient Boosting achieving the highest mean AUROC (0.825 ± 0.010), and SVM showing stable recall across folds (0.799 ± 0.010).

**Table 2:** Evaluation metrics for machine learning models

Model	Accuracy (Test) [95% CI]	Precision (Test)	Recall (Test)	F1-score (Test) [95% CI]	F1-score (CV Mean ± SD)	Recall (CV Mean ± SD)	AUROC (Test) [95% CI]	AUROC (CV Mean ± SD)
LR	0.725 [0.711–0.741]	0.735	0.746	0.740 [0.725–0.757]	0.745 ± 0.010	0.762 ± 0.008	0.794 [0.780–0.811]	0.795 ± 0.010
SVM	0.740 [0.724–0.754]	0.739	0.78	0.759 [0.742–0.775]	0.763 ± 0.008	0.791 ± 0.007	0.796 [0.779–0.810]	0.799 ± 0.010
KNN	0.715 [0.699–0.730]	0.732	0.722	0.727 [0.710–0.743]	0.732 ± 0.012	0.744 ± 0.024	0.762 [0.746–0.779]	0.767 ± 0.011
DT	0.689 [0.672–0.704]	0.727	0.652	0.687 [0.669–0.706]	0.708 ± 0.014	0.677 ± 0.017	0.716 [0.697–0.732]	0.729 ± 0.012
RF	0.709 [0.693–0.723]	0.726	0.716	0.721 [0.704–0.737]	0.735 ± 0.010	0.736 ± 0.018	0.767 [0.751–0.781]	0.781 ± 0.012
GB	0.737 [0.723–0.751]	0.74	0.769	0.755 [0.738–0.769]	0.767 ± 0.009	0.780 ± 0.008	0.817 [0.805–0.831]	0.825 ± 0.010
XGB	0.727 [0.711–0.741]	0.741	0.738	0.739 [0.723–0.755]	0.758 ± 0.011	0.761 ± 0.013	0.800 [0.784–0.814]	0.812 ± 0.010

### Confusion Matrix Analysis

The Confusion Matrix (Table 3) provides a granular view of the classification strengths and weaknesses of each model. From a policy perspective, models that minimize false negatives (e.g., SVM and GB) are particularly valuable because they help identify women who are likely to drop out of institutional delivery despite attending ANC. While the Decision Tree showed a relatively high number of true positives, this was offset by its very high false-negative count (593), indicating inconsistent performance. The DT exhibited lower false positives, but this came at the cost of higher false negatives, limiting its practical utility. DT recorded the highest number of false negatives and the lowest number of true negatives, reinforcing its overall weaker performance.

Conversely, the SVM model demonstrated strong negative classification, achieving the highest number of true negatives (1319) and the lowest number of false negatives (372). Similarly, the GB performed strongly with 1301 True Negatives and 390 False Negatives.

**Table 3:** Confusion matrix summary of model predictions

Confusion Matrix				Models			
	LR	SVM	KNN	DT	RF	GB	XGB
TP	1074	1064	1082	1117	1067	1073	1092
FP	455	465	447	412	462	456	437
FN	430	372	470	593	488	390	443
TN	1261	1319	1221	1098	1203	1301	1248

### Precision–Recall Curve Analysis

**Fig. 4:**
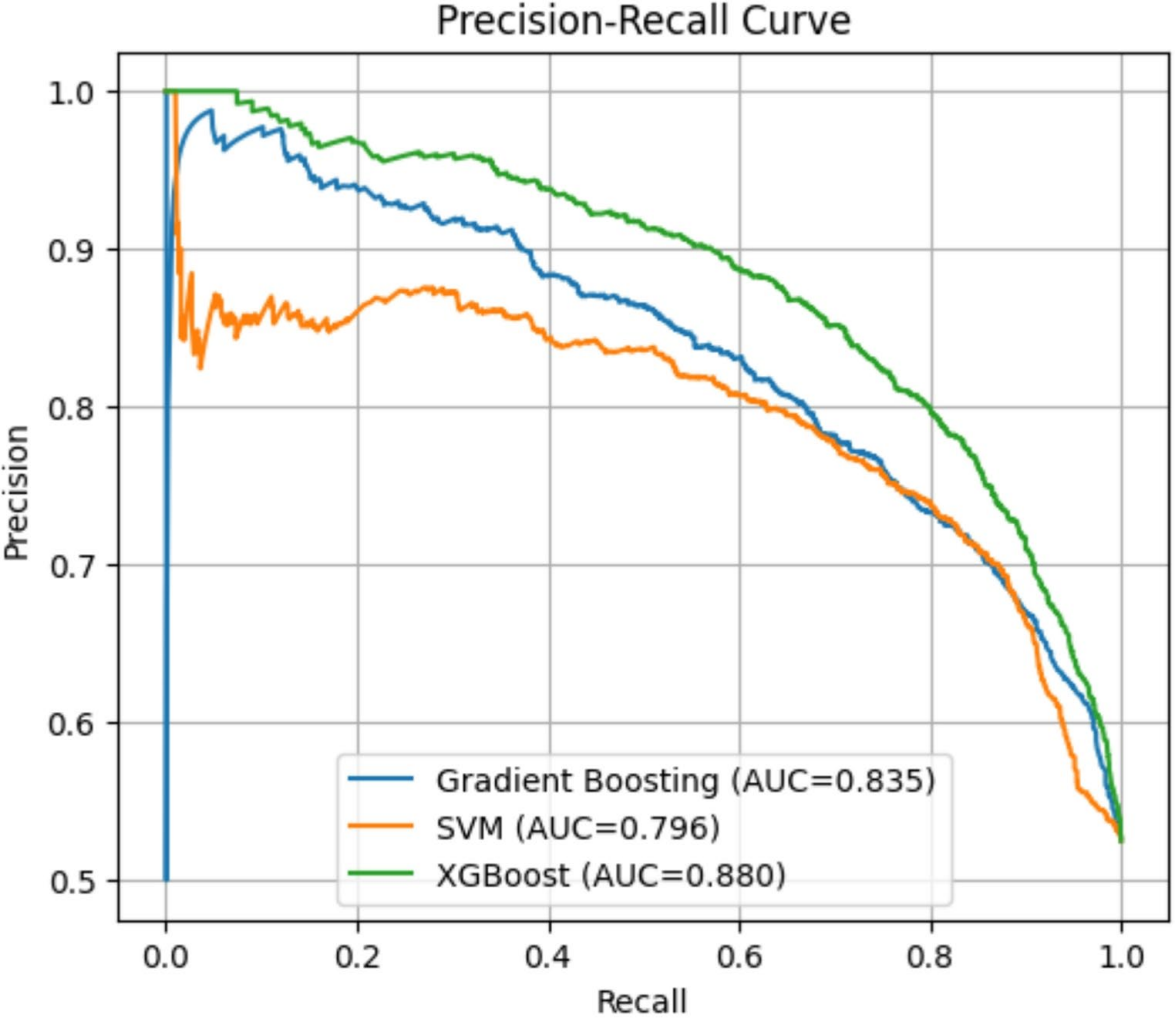
Precision-Recall Curve

Given the moderate class imbalance in the institutional delivery dropout outcome, the precision–recall curves shown in Fig. 4 provide additional insight into the model performance. XGBoost achieved the highest precision–recall AUC (0.880), followed by Gradient Boosting (0.835). The SVM showed a lower precision–recall AUC (0.796), reflecting its slightly reduced precision at higher recall levels, despite its strong sensitivity. These patterns demonstrate that ensemble models, especially XGBoost and Gradient Boosting, maintain better trade-offs between precision and recall across thresholds, which is important for minimizing both false positives and false negatives in operational environments.

### ROC Curve Analysis

**Fig. 5:**
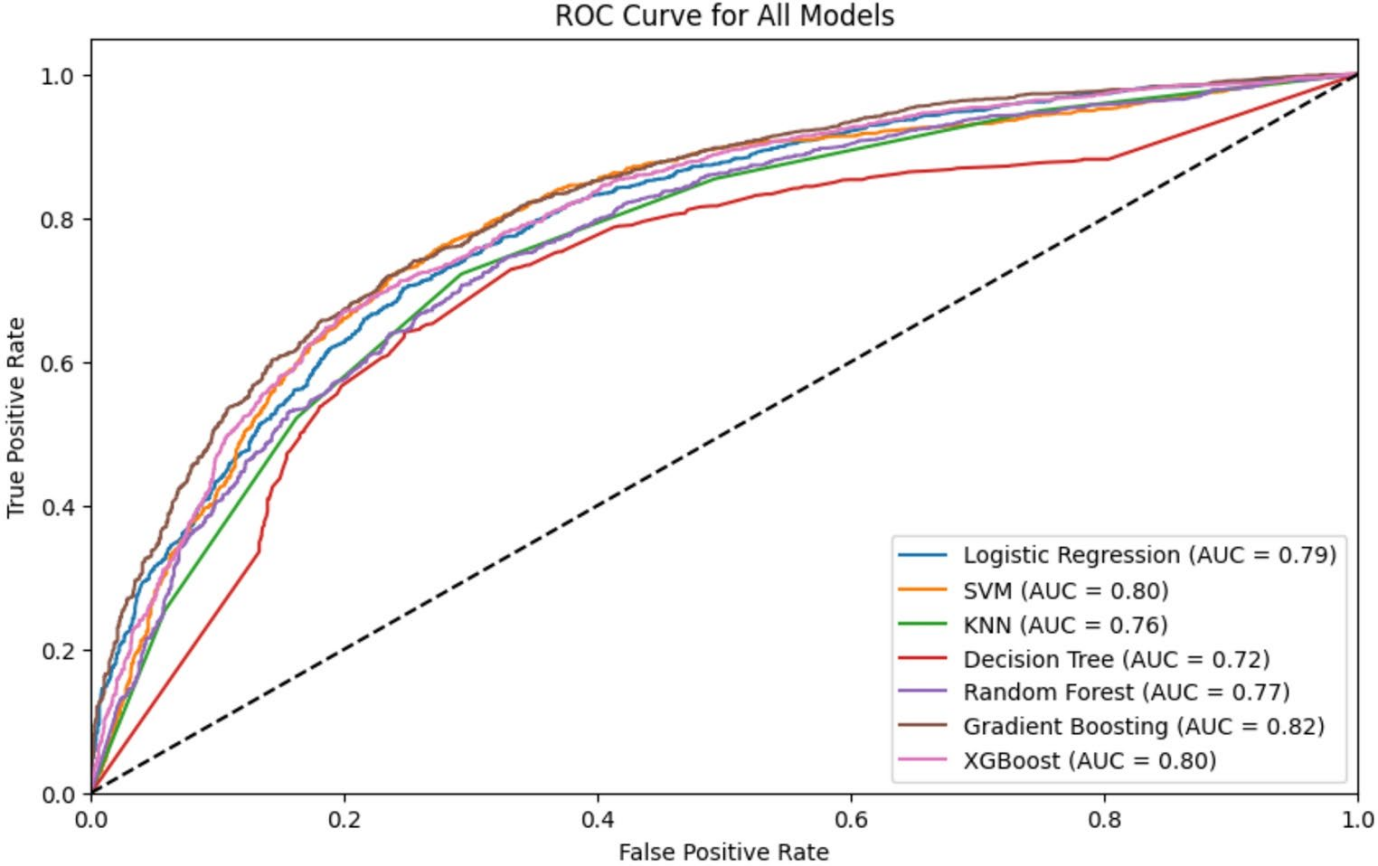
AUROC Curve for all models

The AUROC curve, shown in Fig. 5, provides further insight into each model’s ability to discriminate between institutional delivery dropouts and successful institutional deliveries. GB achieved the highest Area Under the Curve (AUC = 0.82), indicating its superior capability in distinguishing between the two classes. This was closely followed by XGB and SVM, both of which demonstrated strong discrimination, with an AUC of 0.80. In contrast, the DT model exhibited the lowest AUC (0.72), signifying its least effective discrimination between dropout and non-dropout cases. These results reinforce the comparatively stronger performance of Gradient Boosting, XGBoost, and SVM models in predicting institutional delivery dropout.

### Calibration Curve Analysis

**Fig. 6:**
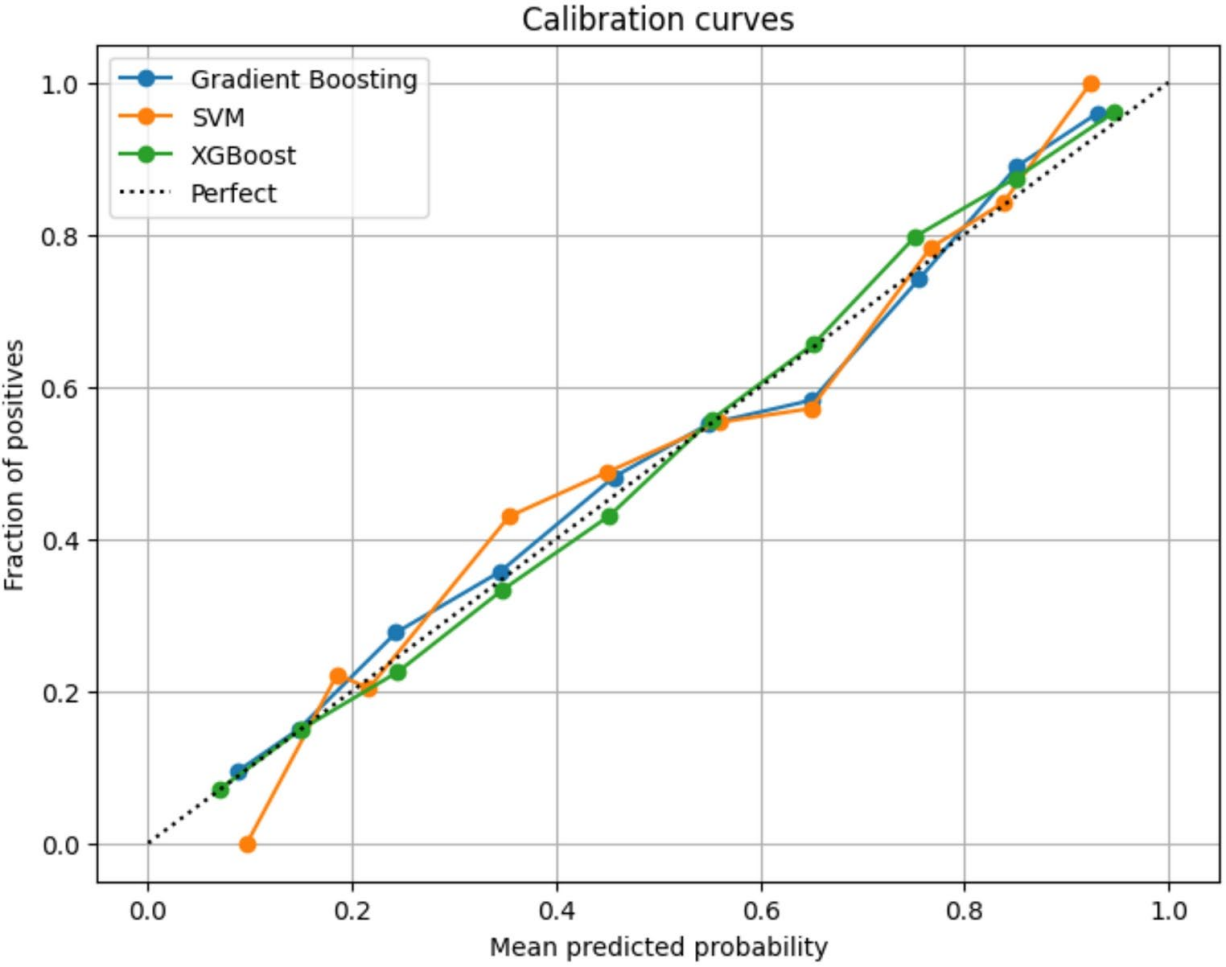
Calibration curve analysis

Figure 6 presents the calibration curves for Gradient Boosting, SVM, and XGBoost. Gradient Boosting demonstrated the closest alignment between predicted probabilities and observed dropout outcomes across the full probability range, indicating the best overall calibration. XGBoost also showed strong calibration, with only mild deviations at mid-range probabilities. The SVM exhibited a slightly greater underestimation at lower predicted probabilities but remained acceptably calibrated. These findings align with the AUROC and F1-score results, further supporting the reliability of the Gradient Boosting and XGBoost models for probability-based risk stratification.

### SHAP-Based Features Importance Analysis

**Fig. 7:**
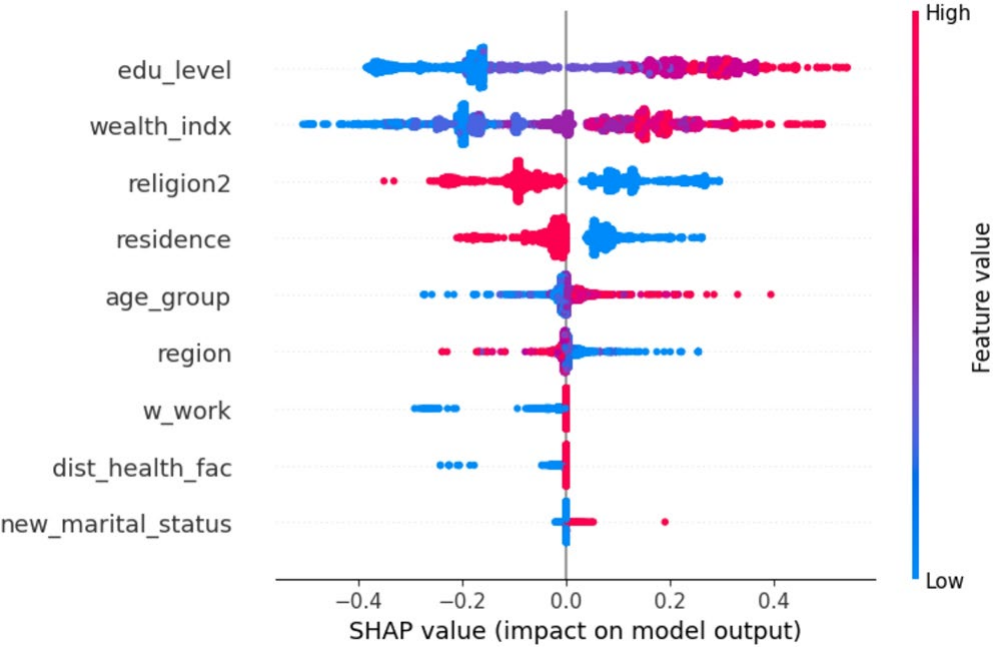
SHAP Summary plot of feature contributions to model predictions

**Fig. 8:**
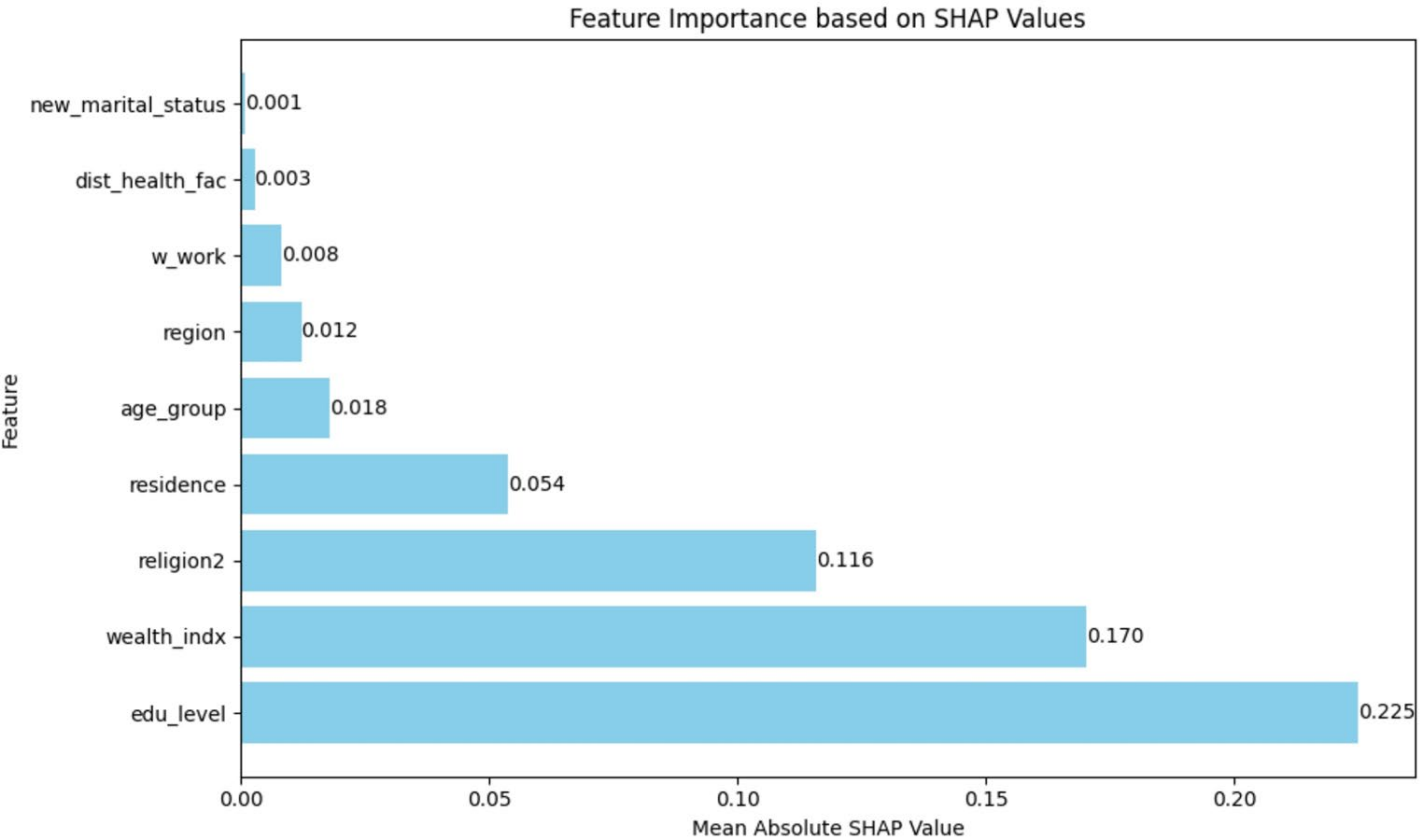
Feature importance ranking based on mean absolute SHAP values

**Fig. 9:**
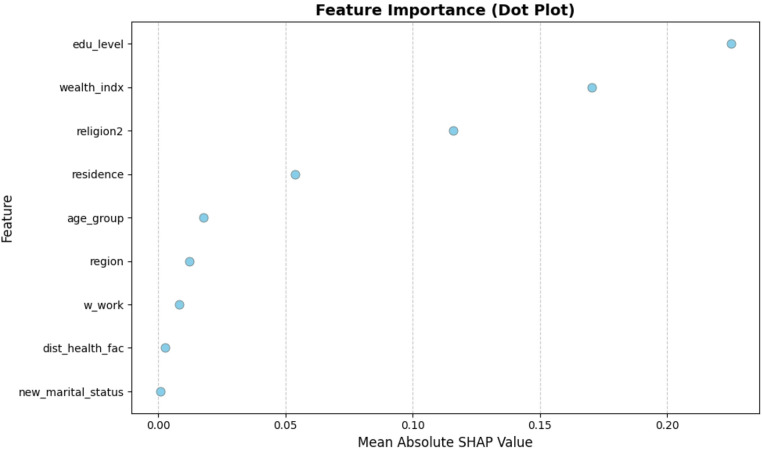
SHAP dot plot of feature importance on model output

The SHAP summary plot (Fig. 7) and importance rankings (Fig. 8) provide critical insights into the features that are most influential in predicting IDD using machine learning models (Fig. 9). Education level remained the strongest predictor, with higher education levels associated with a reduced dropout probability. This was closely followed by the wealth index and religious affiliation, both of which demonstrated substantial impacts on the prediction of IDD. Specifically, higher wealth quintiles and Christian affiliation were generally associated with a lower dropout risk.

Other significant features contributing to the model’s predictions included the place of residence (urban vs. rural), age group, and geopolitical region. These variables consistently showed considerable importance in shaping the model’s output regarding the dropout likelihood. In contrast, marital status was identified as having minimal impact relative to other predictors. These SHAP-based findings largely align with the associations observed in the initial bivariate analysis, reinforcing the robustness of the predictive models and offering interpretable insights that may support the design of targeted interventions in Nigeria. This alignment between the SHAP-derived feature rankings and bivariate COR estimates reinforces the epidemiological validity of the ML models. While odds ratios summarize average effects, SHAP further allows for the exploration of interaction effects and nonlinearities. Although PDP/ICE plots were not included, the SHAP plots revealed patterns such as a declining dropout probability with increasing education or wealth levels, suggesting actionable thresholds for intervention design. Overall, the results support the use of interpretable ensemble ML models, such as GB and SVM, for predicting IDD. These models offer both strong predictive accuracy and transparent, actionable insights that can inform maternal health targeting strategies on a large scale.

### Robustness Check (Sensitivity to Survey Weights)

To assess whether the SHAP-derived feature importance rankings were sensitive to the omission of DHS survey weights in the ML models, a robustness analysis was conducted (Table 4). SHAP rankings were compared with feature rankings obtained from a weighted logistic regression model using the absolute standardized coefficients (β_std). As shown in Table 4, the two ranking systems demonstrated strong concordance, with a Spearman rank correlation of 0.717 (*p* = 0.0298). Importantly, the three highest-ranked predictors–wealth index, education level, and religion–appeared in the same order in both approaches. This indicates that the core interpretive conclusions are stable and not substantially influenced by excluding survey weights from the machine learning models.

**Table 4:** Robustness analysis

Rank	Unweighted ML Model (SHAP Rank)	Weighted Logistic Regression (LR Rank)	Weighted LR (βstd​)	Discrepancy (LR Rank - SHAP Rank)
1	Wealth Index	Wealth Index	0.5238	0
2	Education Level	Education Level	0.5039	0
3	Religion	Religion	0.4321	0
4	Age Group	Residence	0.164	2
5	Region	Working Status	0.0827	-3
6	Residence	Age Group	0.0538	-2
7	Working Status	Distance to Health Facility	0.037	-2
8	Marital Status	Region	0.0365	3
9	Distance to Health Facility	Marital Status	0.0277	1

**Concordance Summary**: *Spearman ρρ = 0.717*,* P-value = 0.0298*

## Discussion

This study leveraged ML techniques augmented by SHAP for model interpretability to investigate IDD among women in Nigeria who accessed ANC. Our findings highlight the utility of ensemble and kernel-based learning approaches in modeling complex maternal health behaviors, with strong predictive performance and interpretable outputs. To our knowledge, this is one of the first studies to apply and benchmark multiple ML algorithms, including ensemble models, alongside logistic regression, to predict institutional delivery dropout using a large, nationally representative dataset from Nigeria. This study extends prior ML applications in maternal health, which have typically focused on early ANC initiation, skilled birth attendance, or fertility preferences (21, 22, 23). In contrast, our study evaluated a broader suite of algorithms and benchmarked ensemble methods and SVM against logistic regression to provide a more comprehensive comparison. This comparative approach underscores the novelty of this study by simultaneously emphasizing both the predictive performance and model interpretability through SHAP integration. Gradient Boosting outperformed all other models across the most critical evaluation metrics, achieving the highest AUROC (0.817) and the highest F1-score (0.755), thereby demonstrating superior overall predictive ability. The strong recall performance of SVM (0.780) further demonstrates meaningful improvements over simpler methods, which often struggle to model nonlinear sociodemographic interactions. Together, these findings show that ensemble learning approaches, especially Gradient Boosting, can capture complex dependencies in sociodemographic predictors that influence dropout after ANC uptake.

Among the seven classifiers evaluated, the SVM demonstrated a strong performance across multiple metrics, achieving the highest recall (0.780) and a competitive F1 score (0.759). However, Gradient Boosting clearly outperformed all models overall, achieving the highest AUROC (0.817), the highest F1 score (0.755), and balanced precision (0.740) and recall (0.769). XGB also performed strongly, achieving the highest precision (0.741) and demonstrating robust discrimination (AUROC = 0.800). The strong performances of GB and SVM highlights their capability to capture subtle nonlinear interactions between predictors, particularly across socioeconomic and regional subgroups. This aligns with prior evidence that ensemble- and kernel-based learners often outperform linear classifiers when the relationships are complex or hierarchical. These results collectively demonstrate the superior accuracy and generalization capability of ensemble methods in complex public health prediction tasks, consistent with prior ML studies (26, 29, 32).

Unlike previous ML studies that applied SHAP to predict antenatal care initiation or fertility preferences (21, 22, 23), this study focused explicitly on the IDD, a distinct and understudied discontinuity in the continuum of care. By comparing seven classifiers and incorporating SHAP, this study contributes to the methodological breadth and contextual relevance of the literature. This focus on ANC-to-delivery discontinuity is critical for health systems seeking to target the most vulnerable cases because the dropout risk remains high even among women who have already engaged with ANC. These insights have actionable value for identifying high-risk women early in the care continuum, thus supporting the design of more adaptive maternal health programs in resource-constrained settings. Conventional models, such as LR and DT, consistently exhibited lower performances across key metrics. DT, in particular, demonstrated the lowest Accuracy (0.688), Recall (0.652), and AUROC (0.716) among all models, alongside the highest number of False Negatives (593) in the confusion matrix, underscoring its limitations in capturing the non-linear interactions and higher-order dependencies present in maternal health service use (30, 33, 34).

The application of SHAP values to interpret model predictions provides granular insight into the contribution of individual features. Education level, wealth index, and religious affiliation consistently appeared as the most influential predictors of institutional delivery dropout, aligning with the strong associations observed in the initial bivariate analysis. These findings reinforce previous research (3, 10, 35, 35, 36) but, more importantly, demonstrate that explainable AI techniques can quantify established social determinants within a predictive modelling framework (18, 21, 37, 38). SHAP also addresses the black-box limitation of many ML models by explaining which predictors drive model decisions and how they influence individual predictions (16, 39, 40). Although PDP or ICE plots were not included, the SHAP values highlighted important nonlinearities and interactions, such as a decreasing dropout probability with increasing education or wealth. These patterns suggest potential thresholds that may be useful for designing targeted interventions. In practice, SHAP-based risk stratification could support targeted counselling during ANC visits, prioritize follow-up by community health workers, and inform geographically tailored outreach strategies aimed at improving continuity of care. While complementary tools could provide further detail, SHAP alone offered sufficient interpretability for this exploratory analysis.

Unlike traditional statistical modelling, which often relies on assumptions of linearity and independent effects, the ML methods employed in this study, particularly boosting algorithms, captured complex interactions across sociodemographic variables (41, 42, 43). SHAP plots further illustrated that the influence of wealth and education varied across geopolitical regions and urban or rural residences, demonstrating interaction effects that are not easily detected in logistic regression. Such interactions are difficult to capture with simpler models, such as logistic regression, further demonstrating the value of ML approaches in maternal health analytics (42, 44, 45, 46). These findings should be interpreted cautiously, as the predictive associationsidentified byythee ML models do not imply causality. This analysis was exploratory and intended to inform risk stratification rather than identify causal pathways. Policy implications should therefore be considered indicative rather than prescriptive.

The integration of the initial bivariate analyses and crude odds ratios served as an important baseline reference. This approach confirmed the consistency of the ML model outputs with traditional epidemiological associations, thereby strengthening confidence in the ML approach by validating that the models were learning from real, epidemiologically plausiblepatternss rather than simply overfitting to noise (47). However, unlike static statistical summaries, the ML pipeline facilitated iterative training, cross-validation, and comparative evaluation of multiple algorithms, providing a scalable and adaptable framework for health systems research (22, 48, 49). These findings suggest opportunities for integrating predictive analytics into maternal health systems in Nigeria. Gradient Boosting and SVM, due to their strong discrimination, sensitivity, and interpretability, could support real time risk alerts during ANC visits. SHAP-informed decision-support dashboards could further enable frontline health workers and program managers to identify women at an elevated risk of dropout and deploy timely, targeted interventions.

### Strengths and Limitations

This study had several strengths that enhanced its rigor and relevance. By applying a comparative machine learning framework incorporating seven commonly used classifiers, this study systematically evaluated and identified the most effective predictive models for IDD. The innovative use of SHAP (SHapley Additive exPlanations) provided transparent and interpretable explanations of the model’s predictions, helping to bridge the gap between predictive performance and actionable insights for policymakers and public health practitioners. Additionally, this study leveraged a large, nationally representative dataset from the 2018 NDHS, which strengthened the generalizability of the findings across Nigeria’s diverse population. Traditional statistical analyses were employed to benchmark machine learning outputs, ensuring that the results were epidemiologically valid and consistent with established associations.

This study has some limitations that should be considered when interpreting its findings. First, the machine learning models were implemented using default hyperparameters without optimization, which may have limited their peak predictive performance. However, this approach reflects the exploratory and benchmarking nature of the study and the fact that it represents one of the first machine learning–based investigations of institutional delivery dropout in Nigeria. Future research may build on this study by applying systematic hyperparameter optimization to enhance the predictive performance in deployment-oriented settings. Second, self-reported obstetric experience variables, such as delivery complications, were excluded because they were inconsistently reported and exhibited substantial missing data. In addition, facility-level characteristics, including staffing levels, service readiness, and objective quality-of-care indicators, were not collected in the DHS household survey and were therefore unavailable for inclusion. As a result, the models may not fully capture the facility-level or supply side determinants of institutional delivery dropout. Third, although STATA 17 was used for descriptive and bivariate epidemiological analyses, formal statistical significance testing of differences in machine learning model performance (e.g., DeLong’s test for AUROC or McNemar’s test for paired classification outcomes) was not performed. This reflects the conceptual distinction between inferential statistical modelling and predictive machine learning. Machine learning evaluation prioritizes predictive accuracy, discrimination, and generalizability rather than hypothesis testing, and uncertainty in model performance is quantified using bootstrapped confidence intervals and cross-validation variability. Consequently, differences in model performance should be interpreted descriptively rather than statistically significant in an inferential sense. Fourth, although stratified five-fold cross-validation was employed to minimize overfitting and improve internal validity, no external validation using regional or temporal partitions was conducted, which may limit the generalizability of the predictive models beyond the analytic sample size. Fifth, SHAP was the only interpretability method used in this study. While SHAP provides robust, model-agnostic explanations of feature contributions and is sufficient for the exploratory scope of this analysis, additional tools such as partial dependence plots or ICE plots could provide complementary insights into feature interactions in future work. Sixth, the NDHS may underrepresent displaced, marginalized, or hard-to-reach populations, which may introduce selection bias and limit the representativeness. Finally, the machine learning models were intentionally not survey-weighted to prioritize predictive performance over population-level inference, which limited the direct interpretability of predicted probabilities as nationally representative estimates.

## Conclusion

This study demonstrated that machine learning models, particularly Gradient Boosting, SVM and XGB, can effectively predict IDD among Nigerian women who have accessed antenatal care. Among all the evaluated models, GB achieved the strongest overall predictive performance, whereas SVM demonstrated the highest sensitivity, and both models provided interpretable outputs through SHAP. The integration of SHAP allowed for the clear identification of high-risk groups based on individual-level sociodemographic characteristics, such as low education, poverty, rural residence, and religious affiliation. These interpretable insights hold practical value for maternal health programming by enabling the early identification of women at risk of dropping out and supporting targeted outreach. Although these results confirm the predictive potential of interpretable machine learning, they remain exploratory. The framework presented in this study can serve as a foundation for the future integration of predictive analytics into Nigeria’s maternal health information systems and community-based outreach platforms. Although the overall accuracy was high for some models, class imbalance required careful interpretation, which was addressed using precision, recall, F1 score, and confusion matrix values. Overall, this study highlights the added value of interpretable machine learning in advancing data-driven and context-specific maternal health interventions in Nigeria and similar low-resource settings.

## Data Availability

No primary data collection was conducted for this study. The analysis was based on secondary data from the 2018 Nigeria Demographic and Health Survey (NDHS), which is publicly available via the DHS Program website: [https://dhsprogram.com](https:/dhsprogram.com) .

## Code Availability

The Python code used for data processing, model development, evaluation, and SHAP analyses is available from the corresponding author upon request.

## References

[1] Afape AO, Azubuike PC, Ibikunle OO, Barrow A. Prevalence and determinants of skilled birth attendance among young women aged 15–24 years in Northern nigeria: evidence from multiple indicator cluster survey 2011 to 2021. BMC Public Health. 2024 Sept;11(1):2471. 10.1186/s12889-024-19976-8 PMC11389318 39256660

[2] Walker T, Woldegiorgis M, Bhowmik J. Utilisation of skilled birth attendant in low-and middle-income countries: trajectories and key sociodemographic factors. Int J Environ Res Public Health. 2021;18(20):10722. 34682468 10.3390/ijerph182010722 PMC8535845

[3] Asefa A, Gebremedhin S, Messele T, Letamo Y, Shibru E, Alano A, et al. Mismatch between antenatal care attendance and institutional delivery in South ethiopia: A multilevel analysis. BMJ Open. 2019;9(3):e024783. 30898814 10.1136/bmjopen-2018-024783 PMC6527994

[4] Theisen OM, Strand H, Østby G. Ethno-political favouritism in maternal health care service delivery: Micro-level evidence from sub-Saharan Africa, 1981–2014. Int Area Stud Rev. 2020;23(1):3–27.

[5] Negero MG. Women’s Use of Quality Maternal Healthcare Services Across the Continuum in Low and Lower-Middle-Income Countries, with a Focus on Ethiopia [Internet]. University of Technology Sydney (Australia); 2023 [cited 2025 June 17]. Available from: https://search.proquest.com/openview/cd32d8aad96337ff848a32a99ba9d364/1?pq-origsite=gscholar&cbl=2026366&diss=y

[6] Adedokun ST, Uthman OA, Bisiriyu LA. Determinants of partial and adequate maternal health services utilization in nigeria: analysis of cross-sectional survey. BMC Pregnancy Childbirth. 2023 June;20(1):457. 10.1186/s12884-023-05712-4 PMC10283328 37340350

[7] Gebremichael SG, Fenta SM. Determinants of institutional delivery in Sub-Saharan africa: findings from demographic and health survey (2013–2017) from nine countries. Trop Med Health. 2021;49(1):45. 34039443 10.1186/s41182-021-00335-x PMC8152346

[8] Kabir S, Hasan MR, Hossain MI, Suraiya S, Islam FB, Nayan MIH et al. Determinants and Trends of Health Facility Delivery in Bangladesh: A Hierarchical Modeling Approach. Souza RT, editor. BioMed Research International. 2022;2022(1):1359572. 10.1155/2022/1359572 PMC9355761 35937411

[9] Tekeba B, Zegeye AF, Gebrehana DA, Tamir TT. Prevalence and determinants of home delivery among women with easy access to health facilities in Sub–Saharan African countries: A Multi–level mixed effect analysis. Ann Glob Health. 2025;91(1):5. 39896101 10.5334/aogh.4615 PMC11784499

[10] Thapa B, Karki A, Sapkota S, Hu Y. Determinants of institutional delivery service utilization in Nepal. PLoS ONE. 2023;18(9):e0292054. 37733812 10.1371/journal.pone.0292054 PMC10513198

[11] Shanmugasundar G, Vanitha M, Čep R, Kumar V, Kalita K, Ramachandran M. A comparative study of linear, random forest and adaboost regressions for modeling non-traditional machining. Processes. 2021;9(11):2015.

[12] Ibikunle O, Usuemerai P, Abass L, Alemede V, Nwankwo E, Mbata A. Artificial intelligence in healthcare forecasting: enhancing market strategy with predictive analytics. Int J Appl Res Social Sci. 2024;6:2409–46.

[13] Malde A, Prabhu VG, Banga D, Hsieh M, Renduchintala C, Pirrallo R. A machine learning approach for predicting maternal health risks in Lower-Middle-Income countries using sparse data and vital signs. Future Internet. 2025;17(5):190.

[14] Margret I, Rajakumar K, Arulalan K, Manikandan S, Valentina. Machine Learning-Based box models for pregnancy care and maternal mortality reduction: A literature survey. IEEE Access. 2024;PP:1–1.

[15] Wang J, Qin Z, Hsu J, Zhou B. A fusion of machine learning algorithms and traditional statistical forecasting models for analyzing American healthcare expenditure. Healthc Analytics. 2024;5:100312.

[16] D’Amore FM, Moscatelli M, Malvaso A, D’Antonio F, Rodini M, Panigutti M, et al. Explainable machine learning on clinical features to predict and differentiate alzheimer’s progression by sex: toward a clinician-tailored web interface. J Neurol Sci. 2025;468:123361. 39724825 10.1016/j.jns.2024.123361

[17] Zhai Y, Zhang Y, Chu Z, Geng B, Almaawali M, Fulmer R, et al. Machine learning predictive models to guide prevention and intervention allocation for anxiety and depressive disorders among college students. Jour Couns Develop. 2025;103(1):110–25.

[18] Hassija V, Chamola V, Mahapatra A, Singal A, Goel D, Huang K, et al. Interpreting Black-Box models: A review on explainable artificial intelligence. Cogn Comput. 2024;16(1):45–74.

[19] Mathew DE, Ebem DU, Ikegwu AC, Ukeoma PE, Dibiaezue NF. Recent emerging techniques in explainable artificial intelligence to enhance the interpretable and Understanding of AI models for human. Neural Process Lett. 2025;57(1):16.

[20] ŞAHiN E, Arslan NN, Özdemir D. Unlocking the black box: an in-depth review on interpretability, explainability, and reliability in deep learning. Neural Comput Applic. 2025;37(2):859–965.

[21] Sani J, Ahmed MM. Machine learning approach in predicting early antenatal care initiation at first trimester among reproductive women in somalia: an analysis with SHAP explanations. Intelligence-Based Med. 2025;11:100252.

[22] Tadese ZB, Nimani TD, Mare KU, Gubena F, Wali IG, Sani J. Exploring machine learning algorithms for predicting fertility preferences among reproductive women in Nigeria. Front Digit Health. 2025;6:1495382. 10.3389/fdgth.2024.1495382 PMC11781225 39886062

[23] Sani J, Ahmed MM, Oluyomi AO. Exploring Machine Learning Algorithms for Predicting Early Antenatal Care Initiation at First Trimester among Reproductive Women in Nigeria. 2024 [cited 2024 Nov 24]; Available from: https://www.researchsquare.com/article/rs-5321613/latest

[24] Onambele L, Guillen-Aguinaga S, Guillen-Aguinaga L, Ortega-Leon W, Montejo R, Alas-Brun R, et al. Trends, Projections, and regional disparities of maternal mortality in Africa (1990–2030): an ARIMA forecasting approach. Epidemiologia (Basel). 2023;4(3):322–51. 37754279 10.3390/epidemiologia4030032 PMC10528291

[25] Oweibia M, Elemuwa CO, Egberipou T, Timighe GC, Peresuodei S, Wilson TR. Maternal and Child Health Trends in Nigeria: A Scoping Review of NDHS 2018 vs. NDHS 2023. medRxiv. 2025;2025–05.

[26] Jadama AF, Toray MK. June. Ensemble Learning: Methods, Techniques, Application. no 2024.

[27] Vasudevan L, Kibria MG, Kucirka LM, Shieh K, Wei M, Masoumi S, et al. Machine learning models to predict risk of maternal morbidity and mortality from electronic medical record data: scoping review. J Med Internet Res. 2025;27:e68225. 40811480 10.2196/68225 PMC12352520

[28] Sani J, Halane S, Ahmed AM, Ahmed MM. Application of machine learning algorithms and SHAP explanations to predict fertility preference among reproductive women in Somalia. Scientific Reports [Internet]. 2025 July;15(1). Available from: 10.1038/s41598-025-04704-y 10.1038/s41598-025-04704-y PMC12277441 40685342

[29] Rahmatinejad Z, Dehghani T, Hoseini B, Rahmatinejad F, Lotfata A, Reihani H, et al. A comparative study of explainable ensemble learning and logistic regression for predicting in-hospital mortality in the emergency department. Sci Rep. 2024;14(1):3406. 38337000 10.1038/s41598-024-54038-4 PMC10858239

[30] Chakraborty C, Bhattacharya M, Pal S, Lee SS. From machine learning to deep learning: advances of the recent data-driven paradigm shift in medicine and healthcare. Curr Res Biotechnol. 2024;7:100164.

[31] Tp K, Ke J, Th M, Rh P, Aj K, Ma C. Performance of a Maternal Risk Stratification System for Predicting Low Apgar Scores. American journal of perinatology [Internet]. 2024 Oct [cited 2025 Oct 20];41(13). Available from: https://pubmed.ncbi.nlm.nih.gov/38301722/10.1055/a-2259-0472 38301722

[32] Mahajan P, Uddin S, Hajati F, Moni MA. Ensemble learning for disease prediction: A Review. Healthcare (Basel). 2023 June 20;11(12):1808. 10.3390/healthcare11121808 PMC10298658 37372925

[33] Hill P, Jonsson D, Lederman J, Bolin P, Vicente V. Uncovering nonlinear patterns in time-sensitive prehospital breathing emergencies: an exploratory machine learning study. BMC Med Inf Decis Mak. 2025 June;3:25:205. 10.1186/s12911-025-03046-z PMC12135243 40462078

[34] Zhou L, Zhu Q, Chen Q, Wang P, Huang H. Predicting hospital outpatient volume using xgboost: a machine learning approach. Sci Rep. 2025;15(1):1–13. 40379678 10.1038/s41598-025-01265-y PMC12084583

[35] Arega T, Mulatu T, Alemayehu A, Mussa I, Dheresa M. Institutional delivery and associated factors among women who gave birth in Benishangul Gumuz region, South West Ethiopia. Front Public Health. 2022;10:965524. 36568776 10.3389/fpubh.2022.965524 PMC9780484

[36] Mohammed S, Worku A, Girma E. Receiving quality antenatal care service increases the chance of maternal use of skilled birth attendants in ethiopia: using a longitudinal panel survey. PLoS ONE. 2022;17(12):e0279495. 36548352 10.1371/journal.pone.0279495 PMC9778501

[37] Band SS, Yarahmadi A, Hsu CC, Biyari M, Sookhak M, Ameri R, et al. Application of explainable artificial intelligence in medical health: A systematic review of interpretability methods. Inf Med Unlocked. 2023;40:101286.

[38] GhoshRoy D, Alvi PA, Santosh KC. Unboxing Industry-Standard AI models for male fertility prediction with SHAP. Healthcare. 2023;11(7):929. 37046855 10.3390/healthcare11070929 PMC10094449

[39] Chinnaraju A, Explainable. AI (XAI) for trustworthy and transparent decision-making: A theoretical framework for AI interpretability. World J Adv Eng Technol Sci. 2025;14(3):170–207.

[40] Hulsen T. Explainable artificial intelligence (XAI): concepts and challenges in healthcare. AI. 2023;4(3):652–66.

[41] Kino S, Hsu YT, Shiba K, Chien YS, Mita C, Kawachi I, et al. A scoping review on the use of machine learning in research on social determinants of health: trends and research prospects. SSM-population Health. 2021;15:100836. 34169138 10.1016/j.ssmph.2021.100836 PMC8207228

[42] Ley C, Martin RK, Pareek A, Groll A, Seil R, Tischer T. Machine learning and conventional statistics: making sense of the differences. Knee Surg Sports Traumatol Arthrosc. 2022;30(3):753–7. 35106604 10.1007/s00167-022-06896-6

[43] Stahl D. New horizons in prediction modelling using machine learning in older people’s healthcare research. Age Ageing. 2024 Sept 23;53(9):afae201. 10.1093/ageing/afae201 PMC11417961 39311424

[44] AlSaad R, Farrell T, Cruz JD, Abd-Alrazaq A, Thomas R, Sheikh J. Artificial intelligence models for predicting the mode of delivery in maternal care. J Gynecol Obstet Hum Reprod. 2025;102976. 10.1016/j.jogoh.2025.102976 40374163

[45] Barnett-Itzhaki Z, Elbaz M, Butterman R, Amar D, Amitay M, Racowsky C, et al. Machine learning vs. classic statistics for the prediction of IVF outcomes. J Assist Reprod Genet. 2020;37(10):2405–12. 32783138 10.1007/s10815-020-01908-1 PMC7550518

[46] Khalil M, McGough AS, Pourmirza Z, Pazhoohesh M, Walker S. Machine learning, deep learning and statistical analysis for forecasting Building energy consumption—A systematic review. Eng Appl Artif Intell. 2022;115:105287.

[47] Moccia C, Moirano G, Popovic M, Pizzi C, Fariselli P, Richiardi L, et al. Machine learning in causal inference for epidemiology. Eur J Epidemiol. 2024;39(10):1097–108. 39535572 10.1007/s10654-024-01173-x PMC11599438

[48] Mahesh B. Machine learning algorithms-a review. Int J Sci Res (IJSR)[Internet]. 2020;9(1):381–6.

[49] Woodman RJ, Mangoni AA. A comprehensive review of machine learning algorithms and their application in geriatric medicine: present and future. Aging Clin Exp Res. 2023;35(11):2363–97. 37682491 10.1007/s40520-023-02552-2 PMC10627901

